# Location Data as Contractual Counter-Performance: A Consumer Perspective on Recent EU Legislation

**DOI:** 10.1007/978-3-662-61920-9_13

**Published:** 2020-06-22

**Authors:** Zohar Efroni

**Affiliations:** 4 MPI for Innovation and Competition, Munich, Germany; 5 MPI for Innovation and Competition, Munich, Germany; 6 MPI for Innovation and Competition, Munich, Germany; 7 MPI for Innovation and Competition, Munich, Germany; Weizenbaum Institute for the Networked Society, Humboldt University Law Faculty, Berlin, Germany

**Keywords:** Location data, Urban mobility, Data as counter-performance, Data protection, Consumer protection, Digital content and services directive

## Abstract

This chapter analyses recent developments in the area of digital consumer law in the EU while focusing on the ‘data as counter-performance’ quandary and its application to location data. The immense technological and economic significance of location data in smart urban spaces renders them a relevant subject for inquiry in the context of ongoing legal efforts to protect consumers who grant permission to use their location data in exchange for digital goods and services.

## Introduction

The classic problem of how to get from point A to point B in the most efficient and convenient way demands new solutions in our digital time and age, especially in modern cities, which are home to 70% of the EU population.^1^ Technological solutions are predominantly based on the generation, collection and extensive use of electronic data. To name just one example, ‘mobility as a service’ (MaaS) stands for a technology-based platform solution in an urban setting that heavily relies on multiple mobility data sources.^2^ Location data play a key role not only in MaaS platforms but also in many other data-driven solutions, technologies, products and business models that shape life in the hyper-connected environment powered by the growth of smartphones. The promise of location-based services and personalised mobility solutions for consumers is considerable—and so are the challenges and risks they pose to individual interests.

A recent privacy incident that has captured much media attention is illustrative. Apple’s iPhone 11 Pro was reported to have continued collecting location data even when the user set the iPhone not to collect such data.^3^ Namely, the phone continued pinging its GPS modules despite users’ deliberate choice to disable this function.^4^ In this way, contrary to users’ expectations and possibly to Apple’s own privacy policy, it was impossible to completely turn off location-based system services simply by individually switching off location services for all applications and system services. Rather, users needed to turn off all global location services in the device settings.

Apple replied to the allegation by explaining that the matter was rooted in the ‘ultra wideband technology’ embedded in the device.^5^ This technology endows the device with spatial awareness to identify other ultra wideband devices nearby. One application of this technology is enabling file sharing between devices via AirDrop.^6^ Apple added that the management of ultra wideband compliance and its use of location data are done entirely on the device and that the company is not collecting user location data.^7^ Still, the revelation was not particularly flattering for a company that takes pride in its comparatively strict privacy and security standards.^8^

The location data that mobile devices collect fuel giant, global and in some cases thinly regulated markets, which often operate and prosper entirely unnoticed by those who own the devices. A series of articles in *The New York Times* picked up the topic. As part of The Privacy Project, reporters obtained a file containing more than 50 billion location pings from over 12 million US citizens as they moved through several major cities such as Washington, San Francisco and Los Angeles.^9^ The newspaper attained the data from a commercial location data company—one of dozens of its kind—that collects precise location data by utilising software included in mobile phone applications. The online article illustrates via the use of interactive heatmaps and analytics techniques how much can be learned about people simply by following their movement traces over time, and how easy it can be to obtain and use such data in the absence of effective regulation.^10^ The report shows further how omnipresent surveillance is and how penetrative it can be. A US advertising executive was quoted as describing the location data industry there as ‘the Wild West’.^11^

Shortly before this chapter went to print, a global crisis overshadowed all the problems location data have elicited so far, and for that matter, it dwarfed all other national, regional and global problems as well: As of July 15th 2020, the novel coronavirus (SARS CoV-2) has caused over thirteen million infection cases and over half a million deaths worldwide. In order to slow down its expansion rate and bring the spread of the pandemic under control, an early identification of infected individuals as well as all other individuals who have been in contact with them is considered critical: Knowing the mobility patterns of positively tested individuals during the relevant period, cross referencing this data with the location data (typically generated by smartphones) of all the persons who were in close physical contact with them, and then, based upon matches, taking preventive measures such as sending direct SMS warnings, ordering quarantine and isolation, conducting pinpointed testing, etc., is considered by many a promising, even a vital strategy to contain the disease.

This current example comes to briefly demonstrate both the enormous utility location data may have and the potential for misuse. In times of crisis such as these, the harm to privacy rights and even to the integrity of the political system in some democracies as a whole often go unnoticed. Less people ponder now whether a massive and unchecked collection of location data by the government as part of the measures it takes against a health disaster of this dimension is justified, proportionate and in conformity with fundamental rights.

In emergency situations, as in normal times, utilising location data is particularly prevalent in modern urban environments, in which mobility becomes ever ‘smarter’ and in which movement patterns can be ascertained and exploited in more accurate, sophisticated and pervasive manners.^12^ With this observation in mind, the aim of this chapter is twofold. The first part (Sect. 2) seeks to sketch the main issues triggered specifically by *location* data and the application of EU data privacy and data protection law to evolving commercial scenarios. This part argues that assessing the problem requires a broad perspective that, besides law, includes technological and economic aspects of newly evolving ecosystems. The three spheres are often intertwined: technological advancements offer new solutions to familiar problems, and moreover, they offer entirely new behavioural options and choices (that might ultimately create new problems). The potential added value for consumers stimulates economic activity and business models designed to monetise technological innovation and enhance consumption. All this happens within a legal environment that might impose restrictions on technology and commerce and where regulative adjustments might be called for.

The second part (Sect. 3) focuses on risks and opportunities for consumers who are willing to trade their (location) data specifically for digital goods and services. Providing the data often relates directly to benefiting from more personalised, finely tuned and, in the end, useful technological solutions. In light of the rising trend often described as (consumer) data commodification,^13^ the second part endeavours to provide initial insights into the problem of location data that economically—and potentially also legally—function as a counter-performance, particularly after the enactment of Directive (EU) 2019/770, which addresses the topic.

## Location Data: Conceptual, Technological and Economic Perspectives

### Conceptual and Definitional Perspectives

#### General Observations

Location data is a term often used in the context of digital technology and economy but which is less often explained or treated as a unique type of data that creates a unique set of problems.^14^ In order to somewhat narrow the scope of the present discussion, it appears reasonable to begin by limiting it to machine-readable data, i.e. data that are generated, stored, analysed, aggregated, enriched, edited, manipulated, transmitted, etc. by the use of digital machines and devices. Next, it is clear that location data in our context go beyond the colloquial meaning of a category of machine-readable data that essentially indicate a physical location in space (often referred to as ‘geolocation’^15^); non-spatial information can also reveal the location of an individual.^16^

In addition, technologies that collect and utilise spatial coordinates very often match it with temporal data, namely timestamps associated with pings of physical locations. The timestamps are an integral element of the data from a technological perspective.^17^ Hence, some academics^18^ and actors in the business-technology sector^19^ use the term spatio-temporal data to more precisely describe the data being collected and processed for analytics, functionality, mobility and other purposes. Moreover, fully capturing the essence and value of location data includes not only an indication of physical location at a certain time but also information about the direction and speed they may encapsulate.^20^

Location data hence provide the basis for *mobility data*,^21^ a concept that is intimately related to the common understanding of smart mobility. In turn, smart mobility was defined on one occasion as ‘collecting, managing, and analysing (fusing) various data sources related to different aspects of residents’ movement in order to better understand and improve the way people move.^22^ It follows that smart mobility crucially depends on high quality mobility data on a massive scale and from multiple sources.

Spatio-temporal data can be said to create an interface layer between the presence and behaviour of a person in cyberspace and the presence and behaviour of that person in real space. Beyond the deductive force of such data (knowing the physical location of a person at a certain time can disclose personal preferences, tastes, behaviours and social connections),^23^ the data interface layer highlights a problem that can be described as the vanishing boundaries between living and operating in these two ostensibly distinct and yet increasingly intertwined spaces.^24^

The location component not only triggers the question of (which) space but also the question of *what* or *whom*. Location data are machine generated. With various levels of accuracy, they ascertain the location of a device—not a natural person. Attributing the location to a specific individual is necessarily based on assumptions, correlations, statistical calculations and often on additional data sets and information that establish the presumed nexus to an individual.^25^ It can be reasonably assumed, for instance, that the location of a smartphone at a certain time and the location of the person registered as its owner are one and the same. Based on device location data alone, however, a certain degree of uncertainty always remains.

#### Legal Definitions

Location data are potentially subject to data protection and data privacy laws. Though the main legal data protection instrument in the EU—the GDPR^26^—mentions location data by name in its definition of ‘personal data’,^27^ it neither defines this term nor provides a detailed explanation. The ePrivacy Directive,^28^ which aims to guarantee the confidentiality of communications over publicly available electronic communication networks and services, defines location data as meaning ‘any data processed in an electronic communications network or by an electronic communications service, indicating the geographic position of the terminal equipment of a user of a publicly available electronic communications service’.^29^ Recital 14 of this Directive is somewhat more detailed in providing that:

Location data may refer to the *latitude, longitude and altitude* of the user’s terminal equipment, to the *direction of travel*, to the level of accuracy of the location information, to the identification of the network cell in which the *terminal equipment is located at a certain point in time* and to the time the location information was recorded (emphasis added).

The ePrivacy Directive distinguishes between ‘location data’ and ‘traffic data’, with the latter defined as ‘any data processed for the purpose of the conveyance of a communication on an electronic communications network or for the billing thereof’.^30^

Based on these definitions, the Directive further distinguishes between the protection scheme and compliance requirements pertaining to ‘traffic data’ on the one hand and ‘location data other than traffic data’ on the other. Regarding the latter category, Art. 9(1) of the ePrivacy Directive provides, *inter alia*, that ‘[w]here location data other than traffic data, relating to users or subscribers of public communications networks or publicly available electronic communications services, can be processed, such data may only be processed when they are made anonymous, or with the consent of the users or subscribers to the extent and for the duration necessary for the provision of a value added service’.

Accordingly, location data only sometimes qualify as traffic data—it depends on whether the data processing goes beyond the mere purpose of enabling the transmission of communication.^31^ This structure, and specifically the lack of sufficient coherence in the distinction between location data that qualify as traffic data and location data that do not as well as the separate sets of rules that apply to each category, has been criticised.^32^ Realising these deficiencies, Art. 29 Working Party (predecessor of the European Data Protection Board) recommended merging the provisions of Art. 6 and Art. 9 of the ePrivacy Directive, suggesting furthermore that both traffic data and location data are ‘metadata’ of increasing informational value that should be subject to a harmonised consent-based regime.^33^

This approach was adopted in the Commission’s proposal for the ePrivacy Regulation,^34^ which, once enacted, would repeal the ePrivacy Directive and drop the distinction between traffic data and location data—including their respective definitions.^35^ At the same time, the ePrivacy Regulation Proposal would introduce an explicit distinction between the content of electronic communications and metadata. Recital 2 of the ePrivacy Regulation Proposal explains:

The content of electronic communications may reveal highly sensitive information about the natural persons involved in the communication, from personal experiences and emotions to medical conditions, sexual preferences and political views, the disclosure of which could result in personal and social harm, economic loss or embarrassment. *Similarly, metadata derived from electronic communications may also reveal very sensitive and personal information.* These metadata includes the numbers called, the websites visited, *geographical location*, the time, date and duration when an individual made a call etc., allowing precise conclusions to be drawn regarding the private lives of the persons involved in the electronic communication, such as their social relationships, their habits and activities of everyday life, their interests, tastes etc.^36^

This approach reflects the understanding that both location data and traffic data fall under the concept of ‘metadata’, a designation that nonetheless is not contradictory to the very sensitive personal information they may contain. The Proposal maintains a different distinction manifested in new definitions of ‘electronic communications content’^37^ and ‘electronic communications metadata’.^38^ Accordingly, data on the location of the device generated in the context of providing electronic communications services and the date, time, duration and type of communication qualify as electronic communications metadata. After noting the great importance users attribute to the confidentiality of their communications and their wish to control the use of electronic communications data for purposes other than conveying the communication, Recital 17 of the Proposal provides:

Therefore, this Regulation should require providers of electronic communications services to obtain end-users’ consent to process electronic communications metadata, which should include data on the location of the device generated for the purposes of granting and maintaining access and connection to the service. *Location data that is generated other than in the context of providing electronic communications services should not be considered as metadata.* Examples of commercial usages of electronic communications metadata by providers of electronic communications services may include the provision of heatmaps; a graphical representation of data using colors to indicate the presence of individuals. To display the traffic movements in certain directions during a certain period of time, an identifier is necessary to link the positions of individuals at certain time intervals. This identifier would be missing if anonymous data were to be used and such movement could not be displayed (emphasis added).

This statement clarifies that location data collected in contexts other than providing electronic communications services^39^ would fall outside the scope of the Regulation. If the same data, however, qualify as personal data under the GDPR, the latter instrument applies and users’ consent might still be required. In the latest iteration and proposed amendments to the text of the ePrivacy Regulation Proposal, introduced by the EU Parliament in late 2019,^40^ an additional Recital (17aa) was proposed:

Metadata such as location data can provide valuable information, such as insights in human movement patterns and traffic patterns. Such information may, for example, be used for urban planning purposes. Further processing for such purposes other than for which the metadata where initially collected may take place without the consent of the end-users concerned, provided that such processing is compatible with the purpose for which the metadata are initially collected, certain additional conditions are met and safeguards are in place, including, where appropriate, the consultation of the supervisory authority, an impact assessment by the provider of electronic communications networks and services and the requirement to genuinely anonymise the result before sharing the analysis with third parties. As end-users attach great value to the confidentiality of their communications, including their physical movements, such data cannot be used to determine the nature or characteristics on an end-user or to build a profile of an end-user, in order to, for example, avoid that the data is used for segmentation purposes, to monitor the behaviour of a specific end-user or to draw conclusions concerning the private life of an end-user. For the same reason, the end-user must be provided with information about these processing activities taking place and given the right to object to such processing.^41^

Overall, the EU legal scheme and recent trends regarding location data are conscious of the increasing utility of location data and the importance of safeguarding users’ privacy and data protection interests, regardless of the specific technology applied. Both the GDPR and the ePrivacy Regulation Proposal advance a technology-neutral approach to their respective subject matters.^42^ In parallel, the conceptual and definitional distinction between content and metadata remains, as does the reliance on anonymisation to reduce risks to privacy interests.

### Technological Perspectives

A myriad of devices and technologies used by urbanites collect, process and exchange location data at a considerable volume, frequency and scale. Location-based services generally aim to obtain the accurate position of individuals—both indoors and outdoors—in order to provide services such as route planning and navigation and to facilitate travel efficiently and comfortably. Global Positioning Systems (GPS) are considered the dominant technology for outdoors positioning as well as the most accurate and reliable, but other technologies are also prevalent, such as WiFi-based localisation cell tower triangulation.^43^ Technologies used for localisation indoors include WiFi (WLAN), internal measurement unit (IMU), radio frequency ID tags (RFID), Bluetooth, GSM and FM.

Research has identified three principal domains in which technology is advancing rapidly, penetration into consumer markets is considerable and location data provide increasing functionality: smartphones, connected cars and the Internet of Things (IoT).^44^ In all of these domains, various location technologies are in use, and the positioning data generated are often infused with other information sources such as geographic information system (GIS) data or real traffic data.

Some technologies are specifically tailor-made for smartphones, e.g. applications with location-based check-in services that enable individuals to share their activity-related choices. In particular, social media applications equipped with check-in functions (such as Facebook or Twitter) provide a vast amount of relevant data that help to determine activity patterns in the context of urban mobility. Among other purposes, such data allow researchers and analytics experts to ascertain individual mobility patterns with growing precision and granularity.^45^

The potential of location data is obviously not limited to social media applications with check-in functions. Mobile phone traces can be used for various purposes, ranging from urban transportation modelling and research^46^ to the creation of personal profiles and targeted advertising by commercial entities^47^ as well as areas beyond commerce such as criminal investigations.^48^ Researchers have noticed that companies also use ultrasonic side channels on mobile devices, usually without the customers being aware of it, in order to determine physical locations and content consumption habits and to follow their movements with applications that permanently ‘listen’ through the device’s built-in microphone to ultrasonic beacons in the background.^49^

Due to the extremely broad use of smart mobile devices for performing daily tasks in urban settings, the location points of a growing number of such devices (and by extension, of their users) are being constantly processed, calculated and transmitted. Researchers determined that it is now dramatically easier to track the location of a huge number of mobile devices, ‘leading to a wealth of information about the mobility of humans, vehicles, devices, and practically anything that can be fitted with a mobile computing device’.^50^ And the density of sensors, signals and reception points—particularly in the city—contributes to the aggregation of very precise, high-quality location data.^51^

Developments in the area of consumer IoT also demonstrate an increasing reliance on location,^52^ and the penetration of IoT into more areas of private and social life contributes to an explosion in the volume, variety, accuracy and quality of processable data.^53^ IoT location data are particularly accurate, which also renders them a particularly valuable, multipurpose source for commercial players, among others.^54^ Researchers have begun to take notice of the possible impacts and risks involved in analysing data sets from IoT devices combined with smart city infrastructure in the context of digital forensics,^55^ among other areas. It would not be exaggerated to say that location data are the lifeblood of smart mobility, and IoT devices are one critical source for such data.

Clearly, connected cars, assisted driving technologies and autonomous vehicles (collectively ‘connected cars’) are another important source.^56^ Modern automobiles also become smarter^57^ and more connected thanks to numerous in-car sensors, on-board computing capacities and an internet connection to external sources. According to one account, connected cars are equipped with on-board computers and embedded mobile broadband as well as dozens of sensors and around 40 microprocessors collecting telematics and driver data. These can produce and then upload to the cloud up to 25 GB of data with every driving hour.^58^ A considerable portion of this data qualifies as location data or is part of the mobility data the car generates.

As indicated by researchers, both the technologies that generate the data and technology-based analytics models (including AI) open up an extremely broad range of use cases for such data:

Mobility data have been used to answer questions such as how people travel between cities and what the patterns are of their daily commute, as well as to predict socioeconomic trends, find relationships in online social networks, identify people’s weight and health status, discover employment patterns, and follow the spread of infectious diseases […]. Models of mobility were used in designing public transportation systems, in taxicab allocation, and in performing crowd-sourcing tasks. In addition, the analysis of mobility patterns leads to a growing field of commercial applications by mobile communication service providers […] as well as by several companies that have already started to provide location-based services analyzing mobile phone location traces.^59^

### Economic Perspectives

There is a close bond between the useful things technology makes possible and the commercial endeavours that monetise and design business models around them. Given the sheer wealth of information advanced technologies and analytics methods currently offer, the economic significance of location data can hardly be overstated.^60^ The data have an enormous commercial value for companies that provide a wide range of products and services and sometimes become a key resource for the firm’s value proposition. As mentioned in a recent study, data can become *the product* (as compared to merely enhancing or augmenting an existing product), with location-based services being an archetypical example.^61^

As a result, personal data are being increasingly commodified,^62^ that is, they are being traded and handled by market participants as a valuable commodity.^63^ To name one prominent example, companies such as HERE provide a plethora of services based on the understanding that ‘the world […] is increasingly powered by location data and technology, enabling people and objects to live, move and interact faster, safer and in a more efficient way than ever before’.^64^ HERE, in which major automotive players currently hold significant shares, provides products and solutions that are centred around the idea that location, described as the ‘data layer of everything’, is the one element that is critical to enabling an ‘autonomous world’.^65^ The HERE Open Location Platform is described as being able to create exhaustive data pools (with data gathered from car sensors, smart city systems and/or other IoT platforms) and thereby offer the opportunity to develop advanced location-based services.^66^

HERE is not alone in discovering the economic potential of commercialising high-quality location data on a massive scale. It competes with other players in an ecosystem where the automotive industry and smart mobility are building on AI-based solutions and where business, innovation, markets and the economy at large are ‘data-driven’.^67^ In China, Navinfo is striving to become ‘the digital brain of intelligent driving with ultraprecise location information and automotive-grade semiconductors for Advanced Driver Assistance Systems (ADAS) and autonomous driving’.^68^ In the realm of location-based services, Foursquare, the company that, as per its own statement, ‘invented the check-in’, now has a product (Pilgrim SDK) that embeds foreground and background location awareness into smartphone applications in order to provide contextual content in real time.^69^ According to an online report from 2018, this company generated over 3 billion visits a month from 105 million locations globally.^70^ Such enormous amounts of location data—in some cases the product that carries the entire business model of commercial enterprises—are being successfully and creatively converted into revenue.

A wide range of business models have emerged in the location data ecosystem, including platform, service, hardware and software providers that initially collect the data from consumers; data brokers that specialise in buying and selling data sets in secondary data markets;^71^ and data-driven technology companies that invent sophisticated methods and models to analyse and extract more insights and commercially valuable information from Big Data. Consequently, new markets emerge in which businesses and users directly and explicitly trade personal-level location information.^72^ In other words, business models in which consumers ‘pay’ with their data are on the rise, and consumer protection law is confronted with completely new situations and problems.

## Recent EU Legislation in the Area of Digital Consumer Protection

### Background

In December 2015, the European Commission published two proposals for directives that would regulate certain aspects concerning contracts for the supply of digital content^73^ and for the online sale of goods.^74^ The proposals triggered a lively debate that continued during the various phases of legislation,^75^ with the European Parliament and the Council of the EU proposing significant changes to the original proposals along the way.^76^ At the conclusion of the trilogue negotiations in March 2019, the texts of both directives received their final form,^77^ followed by the official publication of the directives shortly thereafter.^78^

The debate in recent years has circled around several issues,^79^ including (1) coverage of situations in which the consumer provides data as counter-performance instead of a price for digital content and services and (2) the inclusion of embedded digital content under the protection scheme of the directives (in the current texts of the directives such embedded digital content is referred to as ‘goods with digital elements’). Framework questions such as the explicit inclusion of ‘personal data’ as counter-performance and the simultaneous application of the GDPR triggered an extensive discussion. Another question circled around protection to consumers that ‘passively’ provide personal data instead of a price.

The general aim of the resulting directive concerning digital goods and services (DCSD) is to fully harmonise certain requirements concerning contracts between traders and consumers for the supply of digital content or services (Recital 11 DCSD). It is explicitly designed to harmonise

rules on the conformity of digital content or a digital service with the contract, remedies in the event of a lack of such conformity or a failure to supply and the modalities for the exercise of those remedies, as well as on the modification of digital content or a digital service.^80^ Recitals 12 through 17 lay out a fairly long list of matters in which Member States are not strictly bound by the DCSD. These matters include national rules on the formation, validity, nullity or effects of contracts; the legal nature or classification of the contract; remedies for ‘hidden defects’; and claims against any third party that is not the trader. The debate regarding the proper reach of the DCSD did not focus specifically on location data. The remainder of this chapter seeks to fill this gap.

### Data as Counter-Performance

#### Recognition in the DCSD

The initial Commission's proposal (COM-DCD) included a provision that extended the scope of the Directive to cases where the consumer actively provides, in exchange for digital content, counter-performance other than money in the form of *personal data or any other data*.^81^ After much debate over this issue (including a critical opinion issued by the European Data Protection Supervisor^82^), the Directive now sets forth that consumers who provide *personal data* in exchange for digital content or digital services in principle should benefit from the protections therein.^83^ This provision is subject to two exceptions: (1) when the personal data are provided by the consumer is exclusively processed by the trader for the purpose of supplying the digital content or digital services, or (2) for allowing the trader to comply with legal requirements to which the trader is subject—and in both cases, the trader does not process that data for any other propose.^84^

#### Normative Priority of EU Data Protection and Privacy Law

The DCSD now states generally that in the case of any conflict, the GDPR overrides provisions under the DCSD.^85^ The same applies to conflicts with the e-Privacy Directive (Directive 2002/58/EC).^86^ This priority rule is helpful at least on a formal level for resolving questions of parallel application.^87^ It should help domestic legislatures and courts with the task of applying a certain legal regime in case of discrepancies. Such discrepancies are likely in light of the conceptual and practical overlaps between data protection/privacy law (protecting the individual as a data subject/user) and consumer protection law (protecting potentially the same individual as a consumer). This bright-line rule represents the general understanding that neither contract law in general nor specific consumer protection regulations should derogate from the level of protection persons enjoy under data protection and privacy law. More precisely, Art. 3(8) DCSD provides that consumer protection under the DCSD should be ‘without prejudice’ to the data protection body of law.

#### ‘Passively Provided’ Data

Early proposals suggested a distinction between actively and passively provided data in data-as-counter-performance scenarios. Whereas the COM-DCD referred only to data that are *actively* provided by the consumer,^88^ the Council’s draft would have allowed Member States to extend the application of the directive to passively provided data as well.^89^ Both the Council and the EU Parliament refrained from using the term ‘actively’ within their respective amendments to Art. 3 of the DCD draft. The Council’s draft kept the emphasis on actively provided data while excluding collected metadata (such as IP addresses) or automatically generated content (such as information collected and transmitted by cookies).^90^ By comparison, the Parliament's draft (EP-DCD) would allow for the inclusion of data that is provided passively (e.g. personal data collected by the trader such as IP addresses).^91^ The option of excluding passively provided data from the scope of Art. 3 DCSD has been criticised on several grounds,^92^ including the fact that the distinction between actively and passively provided data could turn fuzzy in certain situations.^93^ Ultimately, the phrase ‘actively provide[s]’ was removed from the final text.

Especially relevant to location data is Recital 25 DCSD, which indicates that ‘metadata’ are not covered by the DCSD unless Member States specifically extend the application of this Directive to such situations.^94^ It follows that data which qualify as ‘metadata’ will trigger protection only if the exchange of such data against digital content/services is specifically recognised under domestic law as a ‘contract’.^95^ At the same time, Recital 24 DCSD clarifies generally that the conclusion of the contract and the provision of the data do not have to happen simultaneously or at any specific proximity of time in order for the DCSD to apply.^96^ This Recital includes the ongoing collocation of data that users upload or create in the course of using the digital content/service, which might, under a certain interpretation, also encompass ‘passive’ data provision situations.^97^

Alas, the DCSD does not provide a definition for the term ‘metadata’.^98^ The examples of metadata it mentions—namely, ‘information concerning the consumer’s device or browsing history’—do not offer a conclusive answer. One important area in which this ambiguity is relevant is the case of cookies. It has been argued, for instance, that cookies that collect data such as browsing history (hence ‘metadata’ that the consumer, strictly speaking, neither uploads nor creates) in exchange for digital goods or services is a situation excluded from DCSD.^99^ Another area that comes to mind, of course, is location data.

#### Application to Location Data

Given that only *personal* data can count as counter-performance,^100^ location data would qualify if (a) it is considered ‘personal data’ under the GDPR and if (b) the data are not exclusively processed by the trader for the purpose of supplying the digital content or digital services.^101^ As to the first condition, in light of the GDPR’s broad definition of ‘personal data’^102^ and the corresponding interpretation by the Court of Justice of the European Union (CJEU),^103^ the exclusion of non-personal data might end up having a marginal impact in practice. It is generally reasonable to assume that non-anonymised location data are more valuable than anonymised data to traders in the B2C sector in terms of allowing pinpointed targeted advertising, refined consumer profile building and individualised pricing models. The first condition nonetheless triggers the general problem of how and where to draw the line between personal and non-personal (including anonymised) data.^104^

The ePrivacy Regulation Proposal suggests that location pings require a device identifier to make them useful in terms of creating heatmaps and ascertaining mobility patterns that are important to the research and development of smart mobility concepts in densely populated cities.^105^ Furthermore, depending on the technology and device at play, consumer location data that are collected automatically often come with ‘build-in identifiers’ such as IP address, device ID and advertiser ID in smartphones. Even when separated from those identifiers, location data are particularly susceptible to re-identification attacks, and within the broader discussion about the sheer feasibility of rendering personal data completely and permanently anonymised, location data present an example in support of arguments that total anonymisation cannot be attained.^106^ The upshot is that location data will almost always qualify as personal data under the GDPR (unless sufficiently anonymised before processing under applicable/acceptable technical and legal standards of anonymisation) and thereby fulfil the first condition.

The second condition calls for a careful assessment. Whether the location data that the consumer provides are processed *exclusively for supplying* the digital content/services in accordance with the DCSD depends largely on the facts and circumstances of the individual case. The assessment will be as complex (or as straightforward) as ascertaining the technical, contractual and practical conditions surrounding the exchange. In addition, obligations under the DCSD’s supply and conformity requirements^107^ and perhaps some other sources external to the contract might be relevant.

This restriction under Art. 3(1) is formulated in a very similar way to Art. 6(1)(b) GDPR, which permits the processing of personal data if ‘processing is necessary *for the performance of a contract* to which the data subject is party or in order to take steps at the request of the data subject prior to entering into a contract’ (emphasis added). At the same time, the GDPR provision is somewhat broader compared to Art. 3(1) DCSD. The latter excludes from the concept of counter-performance the processing of personal data exclusively ‘for the *purpose of supplying the digital content or digital service* in accordance with this Directive’ (emphasis added). It seems that, at least in some cases, processing for the purpose of supply is a specific type of contract performance necessity. Under this interpretation, it is conceivable that Art. 6(1)(b) GDPR might also capture processing that is not directly related to *supplying* the contracted subject matter.

The EDPB opined that ‘Article 6(1)(b) [GDPR] applies where either of two conditions are met: the processing in question must be objectively necessary for the performance of a contract with a data subject, or the processing must be objectively necessary in order to take pre-contractual steps at the request of a data subject’.^108^ In this context, the concept of necessity is applied not strictly under contract law but under data protection (objective) assessment criteria. At the same time, even under such a narrow construction of the legal basis of Art. 6(1)(b) GDPR, it is clear that there is no perfect overlap with Art. 3(1) DCSD. As a result, a valid Art. 6(1)(b) GDPR basis does not exclude *a priori* application of the DCSD, but in practice, processing on this basis will often coincide with situations excluded under Art. 3(1) DCSD. In a legal-economic environment that tolerates the consensual commodification of personal data and simultaneously imposes strict data protection limitations on traders, a successful business model seeking to monetise the data will usually need to rely on processing grounds other than contractual performance necessity, mainly on consent.^109^

Indeed, the importance of users’ affirmative consent in situations where location data are being processed by the trader is expected to increase in light of the CJEU jurisprudence on metadata collected by cookies. In the *Planet49* case, the CJEU ruled that a pre-selected checkbox does not fulfil the requirements of consent.^110^ Active, informed and specific consent is required for using both personal and non-personal data covered under the e-Privacy Directive,^111^ and the user should have a viable option to refuse the implementation of cookies as ‘user consent may no longer be presumed but must be the result of active behaviour on the part of the user’.^112^ Similar to data retrieved via cookies (e.g. IP addresses), location data are often collected in the course of a continuous, automated process inherent to using a connected device. The process runs seamlessly in the background without any affirmative action of users to ‘hand over’ their data and sometimes even without their knowledge.

The prominence of consent is expected to grow under the upcoming ePrivacy Regulation as an important lawful basis of processing ‘electronic communications metadata’.^113^ Already today, consent is the main lawful basis of processing location data that qualify as sensitive data under Art. 9 GDPR. The claim that users often do not actively provide explicit consent to the collection of their (personal) location data poses a major compliance challenge that relates to the more general problem of how to improve the consent process in digital and online settings.^114^

In the final analysis, whether consumers actively provide the personal (location) data or not is of secondary importance, and in any case, it should not impose a technical limitation on the DCSC’s scope. For the opposite conclusion, a convincing normative or economic argument saying that location data provided ‘passively’ call for a lower degree of consumer protection would have to be made. The question of how to reconcile commercial data as counter-performance models with privacy and data protection law and their consent requirements (importantly including Art. 7(4) GDPR) will remain the paramount challenge.

### Embedded Digital Content and IoT

#### Products Bundled with Digital Elements

After many twists and turns on the issue of goods with embedded digital content, the DCSD adopted a new definition for ‘goods with digital elements’, meaning ‘any tangible movable items that incorporate, or are inter-connected with, digital content or a digital service in such a way that the absence of that digital content or digital service would prevent the goods from performing their functions’.^115^ This definition covers what is commonly referred to as IoT devices.^116^ IoT devices connect to the internet via IP addresses, and connectivity is by definition essential for them to perform their functions.^117^

The legal scheme explicitly excludes goods with digital elements from the DCSD while making such goods subject to the Sale of Goods Directive (SGD).^118^ Since the SGD applies solely to sales contracts,^119^ and since its definition of a sales contract does not entertain the concept of data as counter-performance,^120^ goods with digital elements for which the consumer provides data instead of a price are covered neither by the DCSD nor by the SGD. It follows that renting, lending and gratis distribution of a consumer IoT device remains outside of the regulative scope of these directives, unless the transaction for the supply of digital elements can be severed from the transaction concerning the physical good and be treated separately and independently.^121^

This ‘distribution of labour’ between the DCSD and the SGD means that unless the physical component serves merely as a data carrier of digital content, the SGD applies exclusively to sales contracts of goods that include digital elements. The question of whether the digital element in a given case is essential for the good to perform its functions is to be answered, to a large extent, by the terms of the contract itself and the surrounding circumstances. For IoT devices covered by the SGD, the Directive’s protection scheme spreads over the digital components alongside the physical elements. It sets forth specific objective requirements for conformity that are typical to digital content and services, such as the duty to inform the consumer and to supply updates, including security updates that are necessary to keep those goods in conformity.^122^ The SGD, however, does not include a detailed provision comparable to Art. 19 DCSD regarding modifications in the digital content or services and the consumer protection safeguards therein.^123^

#### Application to Location Data

The application of the coverage question to IoT devices is certainly relevant for smart mobility. The consumer devices used for smart mobility usually qualify as goods with digital elements under the DCSD/SGD scheme. Those devices rely on location data and connection to the internet is essential for their proper function and utility. During their operation, they establish connection to remote services that access their location data. As noted, in the absence of transfer of ownership for a price, the consumer protection layer of the DCSC/SGD does not apply. It appears that traders still sell most consumer IoT devices for money.^124^ But a shift to business models that more intensively and transparently monetise personal data collected by the device for a considerable discount, a subscription model and/or gratis distribution instead of sales transactions do not seem that farfetched.

Particularly in the consumer IoT and smartphone segments, consumers have a strong incentive to share their location with hardware, software, service and platform providers. Depending on the particular case, sharing location data can dramatically increase personal usability and functionality. The mission of consumer protection law at this juncture should be to ensure that consumers, who suffer from information asymmetry vis-à-vis traders, weaker bargaining positions and in some cases total lack of both bargaining power and viable alternatives, are not being exploited. One important element is imposing transparency obligations on traders to enhance consumers’ understanding of the context, purposes, implications and risks associated with sharing location.

A comprehensive evaluation of the legal position of EU consumers in the IoT segment should include further regulative instruments, such as the Consumer Rights Directive (2011/83/EU) as recently revised by Directive (EU) 2019/2161 (Consumer Rights Modernisation Directive CRMD).^125^ The CRD generally secures broad information rights under Article 5 thereof (including information about the *total price* of the goods or services) as well as specific information requirements for distance or off-premises contracts (Article 6). The revised CRD (to be transposed in national laws by 28 May 2022) borrows many important definitions from the GDPR and the DCSD/SGD scheme.^126^ It will apply explicitly ‘where the trader supplies or undertakes to supply digital content which is not supplied on a tangible medium or a digital service to the consumer and the consumer provides or undertakes to provide personal data to the trader’.^127^

In principle, CRD rights should apply to contracts regarding IoT goods, namely, both to the physical component of the device and the digital content or service that makes it work. But this is not always the case. For instance, some consumer rights specifically attach requirements concerning pre-contractual information duties^128^ or the rights of consumers in the case of withdrawal^129^ to digital content. Under the revised CRD, these rights will also apply to digital content/services of goods with digital elements subject to a sales contract, except for cases where the digital content is supplied on a tangible medium and the consumer ‘pays’ with personal data. This structure suggests that pre-installed digital content on an IoT device does not benefit from the CRD’s protections that apply to digital content.

The synopsis sketched above, while only briefly touching upon the genuinely complex matrix of digital consumer protection law in the EU, demonstrates that the implications of the revised CRD for IoT consumers are not easy to pin down. As the consolidated body of consumer protection law emerging under the New Deal for Consumers Initiative of the European Commission and the enactment of the DCSD/SGD becomes more intricate, the exposition, implementation and compliance challenges are likely to increase and provide fertile ground for further research and discussion.

## Conclusion

Location data remain an extremely relevant and dynamic playing field for technology developers, market actors and consumers. As such, they calls for the attention of lawmakers and courts as they come to define the legal boundaries for these dynamics and, to some extent, prescribe the rules of the game. The task of enabling market models with an increasing reliance on data and their consensual exchange in B2C markets and, at the same time, preserving the rights of individuals *as data subjects and consumers* should not be underestimated. Many questions within data protection and privacy law itself as well as questions concerning its interface with other legal domains such as consumer protection and contract law remain unresolved.

Location data, due to their unique significance and role in the digital economy, could play a pivotal role in the process of figuring out this interplay—which is hopefully moving towards a coherent and consistent legal scheme that finds the right balance between personal autonomy, state intervention and market economy. On the one hand, utilising location data is indispensable for numerous technological innovations and key for economic growth. On the other hand, such utilisation poses new risks to individual interests. Whether location data therefore could and should be treated as a unique category of data from a legal perspective is a vexing question that has not yet been extensively discussed, but it certainly deserves some deeper deliberations.

## Footnotes

1 EU Commission, ‘Urban Mobility Package’ (*European Commission*, 26 August 2020) <https://ec.europa.eu/transport/themes/clean-transport-urban-transport/urban-mobility/urban-mobility-package_en> accessed 26 August 2020.

2 See eg Warwick Goodall and others, ‘The Rise of Mobility as a Service: Reshaping how Urbanites Get Around’ (2017) 20 Deloitte Review 112-129. In this review, MaaS is described as a model which, at its core, relies on a digital platform that integrates end-to-end trip planning, booking, electronic ticketing and payment services across all modes of transportation, public or private, ibid 114. For further analysis and the regulative dimensions of MaaS, see Yanying Li, ‘The Role of Public Authorities in the Development of Mobility-as-a-Service’, in Matthias Finger and Maxime Audouin (eds), *The Governance of Smart Transportation Systems: Towards New Organizational Structures for the Development of Shared, Automated, Electric and Integrated Mobility* (Springer 2019).

3 Kate O’Flaherty, ‘Apple’s iPhone 11 Pro Collects Location Data, Even When Told Not To’ (*Forbes*, 4 December 2019) <www.forbes.com/sites/kateoflahertyuk/2019/12/04/apple-iphone-11-iphone-11-pro-location-privacy-issue> accessed 21 February 2020.

4 ibid: An IT security expert showed how GPS data are also collected when individual location services are disabled in the iPhone 11 Pro’s settings. This happened even when users set their location services toggle to ‘Never’.

5 Zack Whittaker, ‘Apple Says its Ultra Wideband Technology is Why Newer iPhones Appear to Share Location Data, Even When the Setting is Disabled’ (*TechCrunch*, 5 December 2019) <https://techcrunch.com/2019/12/05/apple-ultra-wideband-newer-iphones-location> accessed 21 February 2020.

6 Apple explained: ‘Ultra wideband technology is an industry standard technology and is subject to international regulatory requirements that require it to be turned off in certain locations […]. iOS uses Location Services to help determine if an iPhone is in these prohibited locations in order to disable ultra wideband and comply with regulations.’, ibid.

7 ibid: According to Apple, a new, dedicated toggle option for this feature will be included in upcoming iOS updates.

8 See Apple’s Privacy Governance Statement explaining its cross-functional approach to privacy governance, <www.apple.com/legal/privacy/en-ww/governance> accessed 21 February 2020: ‘At Apple we design our products and services according to the principle of privacy by default and collect only the minimum amount of data necessary to provide our users with a product or service. We also deploy industry-leading consent mechanisms to allow our customers to choose whether to share data such as their Location, Contacts, Reminders, Photos, Bluetooth Sharing, Microphone, Speech Recognition, Camera, Health, HomeKit, Media & Apple Music and Motion & Fitness Data with apps.’

9 Stuart A Thompson and Charlie Warzel, ‘Opinion – The Privacy Project: Twelve Million Phones, One Dataset, Zero Privacy’ *The* *New York Times* (19 December 2019) <www.nytimes.com/interactive/2019/12/19/opinion/location-tracking-cell-phone.html> accessed 21 February 2020.

10 The report notes: ‘You’ve probably never heard of most of the [data brokerage] companies—and yet to anyone who has access to this data, your life is an open book. They can see the places you go every moment of the day, whom you meet with or spend the night with, where you pray, whether you visit a methadone clinic, a psychiatrist’s office or a massage parlor.’, ibid.

11 Stuart A Thompson and Charlie Warzel, ‘Opinion – The Privacy Project: Smartphones are Spies. Here’s Whom They Report To’ *The* *New York Times* (20 December 2019) <www.nytimes.com/interactive/2019/12/20/opinion/location-tracking-smartphone-marketing.html> accessed 21 February 2020.

12 See Jonathan Andrew, ‘Challenges to Locational Privacy: The Transformation of Urban Mobility’, in this volume.

13 See eg Stacy-Ann Elvy, ‘Commodifying Consumer Data in the Era of the Internet of Things’ (2016) 59 Boston College Law Review 424; McKinsey & Company, ‘Monetizing Car Data: New Service Business Opportunities to Create New Customer Benefits’ (*Advanced Industries*, September 2016) <www.mckinsey.com/~/media/McKinsey/Industries/Automotive%20and%20Assembly/Our%20Insights/Monetizing%20car%20data/Monetizing-car-data.ashx> accessed 21 February 2020.

14 One exception is a paper by Keßler and McKenzie in which the authors argue that, at least in the context of privacy, ‘spatial is special’ since information about an individual’s location is substantially different from other kinds of personally identifiable information. See Carsten Keßler and Grant McKenzie, ‘A Geoprivacy Manifesto’ (2018) 22 Transactions in GIS 3, 5ff.

15 See eg the definition of ‘Geolocation’ in Wikipedia <https://en.wikipedia.org/wiki/Geolocation> accessed 21 February 2020.

16 See Keßler and McKenzie (n 14) 10. Sources of non-spatial public or otherwise shared data can reveal the location of an individual by inferring it from information on stated interests, social activities and temporal behaviours.

17 For further discussion, see Jonathan Andrew, ‘Location Data and Human Mobility: An Evaluation of a Dissonance that Frames Data Protection and Privacy Rights’ (PhD Thesis, European University Institute 2018) 32 <https://cadmus.eui.eu/handle/1814/51585> accessed 21 February 2020.

18 ibid 280-281: ‘the term “location data” […] fails to connote a core dimension of the data inhered i.e. the temporal data. A more appropriate nomenclature would be that of ‘spatio-temporal data.’

19 See eg Hasso-Plattner-Institut, ‘Spatio-Temporal Data Analysis’ (*Hasso-Plattner-Institut*) <https://hpi.de/plattner/projects/spatio-temporal-data-analysis.html> accessed 21 February 2020 (references to development projects in the area of time-stamped data analytics); omni.sci, ‘Spatiotemporal Definition’ (*omni.sci*) <www.omnisci.com/learn/resources/technical-glossary/spatial-temporal> accessed 21 February 2020.

20 See eg Uber’s statement that ‘[i]n order to calculate speeds we use two data inputs: (a) GPS locations of vehicles over time, and (b) map data that represents the street network on which vehicles travel’, in ‘Uber Movement: Speeds Calculation Methodology’ <https://movement.uber.com/_static/56b3b1999eb80fadffbeb9bebe9888a7.pdf> accessed 21 February 2020. The PECR in the UK define in s 2(1) location data as specifically including the direction of travel and the time the location information was recorded, <www.legislation.gov.uk/uksi/2003/2426/contents/made> accessed 21 February 2020.

21 See eg Mohamed Maouche, Sonia Ben Mokhtar and Sara Bouchenak, ‘HMC: Robust Privacy Protection of Mobility Data against Multiple Re-Identification Attacks’ (2018) 2(3) Proceedings of the ACM on Interactive, Mobile, Wearable and Ubiquitous Technologies 1, 2. In applying various models, raw location traces are associated with semantic information to yield mobility patterns.

22 Billy Pik Lik Lau and others, ‘A Survey of Data Fusion in Smart City Applications’ (2019) 52 Information Fusion 357, 365. In this article, the authors propose that the smart human mobility domain can be divided into three sub-domains: (1) Smart Location-Based Services, (2) Human Mobility Understanding and (3) Smart Transportation Systems, ibid 365-366.

23 Data logs about past locations combined with social and other data can tell third parties much about the person’s personality, background, preferences and habits. They also have predictive force: based on past location data and an analysis of recurring patterns, informed assumptions can be made regarding the physical location of the person at a future point in time. See eg Ourania Kounadi, Bernd Resch and Andreas Petutschnig, ‘Privacy Threats and Protection Recommendations for the Use of Geosocial Network Data in Research’ (2018) 7 Social Sciences 191.

24 For the argument that software, through its ability to do work in the world, transduces space, see Rob Kitchin and Martin Dodge, *Code/Space: Software and Everyday Life* (MIT Press 2011). For a legal analysis of virtual reality and augmented reality aspects, see Mark Lemley and Eugene Volokh, ‘Law, Virtual Reality, and Augmented Reality’ (2018) 166 University of Pennsylvania Law Review 1051. See also Jannice Käll, ‘Governing Smart Spaces through Autonomous Vehicles’, in this volume.

25 *United States v. Jones*, 565 U.S. 400 (2015). The location of a suspect in a criminal investigation was determined based on GPS location data from a vehicle registered to his wife and on information showing that the wife turned the car over to the suspect for his exclusive use.

26 Parliament and Council Regulation (EU) 2016/679 of 27 April 2016 on the protection of natural persons with regard to the processing of personal data and on the free movement of such data, and repealing Directive 95/46/EC (General Data Protection Regulation) [2016] OJ L119/1 (GDPR).

27 GDPR, art 4(1).

28 Parliament and Council Directive 2002/58/EC of 12 July 2002 concerning the processing of personal data and the protection of privacy in the electronic communications sector (Directive on privacy and electronic communications) [2002] OJ L201/37 (ePrivacy Directive), as amended, *inter alia*, by Parliament and Council Directive 2009/136/EC of 25 November 2009 amending Directive 2002/22/EC on universal service and users’ rights relating to electronic communications networks and services, Directive 2002/58/EC concerning the processing of personal data and the protection of privacy in the electronic communications sector and Regulation (EC) No 2006/2004 on cooperation between national authorities responsible for the enforcement of consumer protection laws [2009] OJ L337/11 (the so-called ‘cookie Directive’).

29 ePrivacy Directive, art 2(c).

30 ePrivacy Directive, art 2(b).

31 Recital 35 of the ePrivacy Directive provides: ‘In digital mobile networks, location data giving the geographic position of the terminal equipment of the mobile user are processed to enable the transmission of communications. Such data are traffic data covered by Article 6 of this Directive. However, in addition, digital mobile networks may have the capacity to process location data which are more precise than is necessary for the transmission of communications and which are used for the provision of value-added services such as services providing individualised traffic information and guidance to drivers. The processing of such data for value added services should only be allowed where subscribers have given their consent. Even in cases where subscribers have given their consent, they should have a simple means to temporarily deny the processing of location data, free of charge.’

32 See eg Andrew, ‘Location Data and Human Mobility’ (n 17) 62-68.

33 Article 29 Data Protection Working Party, ‘Opinion 03/2016 on the evaluation and review of the ePrivacy Directive (2002/58/EC)’ (2016) WP240, 13-16.

34 Commission, ‘Proposal for a Regulation of the European Parliament and of the Council concerning the respect for private life and the protection of personal data in electronic communications and repealing Directive 2002/58/EC (Regulation on Privacy and Electronic Communications)’ COM (2017) 10 final.

35 ibid art 4 of the Proposal, titled ‘Definitions’, no longer contains separate definitions for location data and traffic data.

36 ibid recital 2 (emphasis added).

37 ibid art 4(3)(b): ‘“Electronic communications content” means the content exchanged by means of electronic communications services, such as text, voice, videos, images, and sound.’

38 ibid art 4(3)(c): ‘“Electronic communications metadata” means data processed in an electronic communications network for the purposes of transmitting, distributing or exchanging electronic communications content; including data used to trace and identify the source and destination of a communication, data on the location of the device generated in the context of providing electronic communications services, and the date, time, duration and the type of communication.’

39 For a definition of ‘electronic communications service’, the Proposal refers to art 2(4) of Parliament and Council Directive (EU) 2018/1972 of 11 December 2018 establishing the European Electronic Communications Code [2018] OJ L321/36.

40 Council of the European Union, Proposal for a Regulation of the European Parliament and of the Council concerning the respect for private life and the protection of personal data in electronic communications and repealing Directive 2002/58/EC (Regulation on Privacy and Electronic Communications), 13808/19, Brussels, 8 November 2019.

41 ibid recital 17aa.

42 GDPR, recital 15; ePrivacy Regulation Proposal, recital 14.

43 Lau and others (n 22) 365-366.

44 The distinction between the three domains is by no means clear-cut. This classification, however, is not material for the arguments in this chapter and is offered for convenience only. cf European Data Protection Supervisor (EDPS), ‘TechDispatch #3: Connected Cars’ (*EDPS*, 20 December 2019) <https://edps.europa.eu/data-protection/our-work/publications/techdispatch/techdispatch-3-connected-cars_en> accessed 21 February 2020. This report classifies connected cars as an IoT category and describes the modern car as a ‘computer on four wheels’.

45 Samiul Hasan, Xianyuan Zhan and Satish V Ukkusuri, ‘Understanding Urban Human Activity and Mobility Patterns Using Large-scale Location-based Data from Online Social Media’ in Yu Zheng, Steven E Koonin and Ouri E Wolfson (eds), *UrbComp '13: Proceedings of the 2nd ACM SIGKDD International Workshop on Urban Computing* (Association for Computing Machinery 2013).

46 Francesco Calabrese and others, ‘Understanding Individual Mobility Patterns from Urban Sensing Data: A Mobile Phone Trace Example’ (2013) 26 Transportation Research Part C: Emerging Technologies 301. This study concludes that mobility phone traces represent a reasonable proxy for individual mobility and can provide useful insights into inter-urban mobility patterns under certain conditions.

47 Taylor Peterson, ‘Location Data 101: A Primer for Marketers’ (*Marketing Land*, 19 August 2019) <https://marketingland.com/location-data-101-a-primer-for-marketers-265395> accessed 21 February 2020.

48 *Carpenter vs. United States*, 585 U.S. ___,1-2 (2018). Cell Site Location Information (CSLI) retained by cellular networks was used by investigators to ascertain the location of the suspect at the time of the crime.

49 Daniel Arp and others, ‘Privacy Threats through Ultrasonic Side Channels on Mobile Devices’ in *2017 IEEE European Symposium on Security and Privacy (EuroS&P)* (IEEE 2017) 35–47.

50 Eran Toch and others, ‘Analyzing Large-scale Human Mobility Data: a Survey of Machine Learning Methods and Applications’ (2019) 58 Knowledge and Information Systems 501, 502.

51 *Carpenter vs. U.S.* (n 48) 2: ‘The greater the concentration of cell sites, the smaller the coverage area. As data usage from cell phones has increased, wireless carriers have installed more cell sites to handle the traffic. That has led to increasingly compact coverage areas, especially in urban areas.’

52 See ‘Location Data: IoT Applications and Benefits’ (*IoT for all*, 12 October 2018) <www.iotforall.com/location-data-iot-applications-and-benefits/> accessed 21 February 2020: ‘Location data is how many modern businesses make sense of their processes, their products and/or services, and how people interact with all of the above. It enables businesses to track assets across oceanic black holes. It allows them to map customer journeys seamlessly. It is the tool they use to optimize the routes of swarms of vehicles weaving through smart cities.’

53 Katarzyna Kryla-Cudna, ‘Consumer contracts and the Internet of Things’ in Vanessa Mac, Eric Tjong Tjin Tai and Anna Berlee (eds), *Data Science and Law* (Edward Elgar 2018) 83-86.

54 cf Andrew, ‘Challenges to Locational Privacy’ (n 12) 7-8. See also, Andrew, ‘Location Data and Human Mobility’ (n 17) 179ff. Here the developments in the IoT area are described as a ‘paradigm shift’ in the significance of location data from a legal perspective.

55 Michael M Losavio and others, ‘The Internet of Things and the Smart City: Legal Challenges with Digital Forensics, Privacy and Security’ (2018) 1 Security and Privacy <https://onlinelibrary.wiley.com/doi/epdf/10.1002/spy2.23> accessed 21 February 2020.

56 For simplicity, the term ‘connected cars’ is used while being conscious of the various levels of vehicular automation and connectivity. See eg the explanation of the five automation levels from 1 (driver assistance) to 5 (full automation, driverless car) on the BMW website <www.bmw.com/en/automotive-life/autonomous-driving.html> accessed 21 February 2020.

57 See eg Sylvia Zhang, ‘Who Owns The Data Generated By Your Smart Car?’ (2018) 32 Harvard Journal of Law & Technology 299, 302. This study applies a broad definition to smart car to include any personal vehicle that has connectivity to the Internet, other devices, or surrounding vehicles or infrastructure, and is equipped with external or internal sensors and a method of recording data.

58 Gabriel Seiberth and Wolfgang Gründiger, ‘Data-Driven Business Models in Connected Cars, Mobility Services & Beyond’ (2018) BVDW Research No. 01/18, 21 <www.bvdw.org/fileadmin/user_upload/20180509_bvdw_accenture_studie_datadrivenbusinessmodels.pdf> accessed 21 February 2020 (citing Jorge L Balcells, ‘Datacentres: In the Driving Seat of the Connected Car Revolution’ (*IoT Tech News*, 16 December 2016) <www.iottechnews.com/news/2016/dec/16/datacentres-driving-seat-connected-car-revolution/> accessed 21 February 2020).

59 Toch and others (n 50) 502 (citations omitted).

60 For a recent account on valuation of data in data-driven markets, see Dan Ciuriak, ‘Unpacking the Valuation of Data in the Data-Driven Economy’ (Notes for Remarks at the NYU Conference on Global Data Law, New York, 26-27 April 2019) <https://papers.ssrn.com/sol3/papers.cfm?abstract_id=3379133> accessed 21 February 2020.

61 Seiberth and Gründiger (n 58) 6-7.

62 See eg Sebastian Sevignani, ‘The Commodification of Privacy on the Internet’ (2013) 40 Science and Public Policy 733–739.

63 ibid: Sevignani refers to commodification as ‘the process of making things exchangeable on markets, either actually and/or discursively by framing things as if they were exchangeable’.

64 See statement on HERE’s website <www.here.com/vision> accessed 21 February 2020.

65 ibid.

66 Seiberth and Gründiger (n 58) 36.

67 See Commission, ‘Towards a Thriving Data-Driven Economy’ COM (2014) 442 final.

68 Seiberth and Gründiger (n 58) 12.

69 See Foursquare website <https://enterprise.foursquare.com/products/pilgrim> accessed 21 February 2020.

70 Jordan Crook, ‘Foursquare is Finally Proving its (Dollar) Value’ (*Tech Crunch*, 19 January 2018) <https://techcrunch.com/2018/01/19/foursquare-is-finally-proving-its-dollar-value> accessed 21 February 2020.

71 See Mathew Crain, ‘The Limits of Transparency: Data Brokers and Commodification’ (2016) 20 New Media and Society 88-104.

72 McKinsey & Company (n 13) 13ff. Survey results regarding car data show that consumers are increasingly willing to share data but that they also expect a fair value in return.

73 Commission, ‘Proposal for a Directive of the European Parliament and of the Council on certain aspects concerning contracts for the supply of digital content’ COM (2015) 634 final (hereinafter referred to as ‘COM-DCD’).

74 Commission, ‘Proposal for a Directive of the European Parliament and of the Council on certain aspects concerning contracts for the online and other distance sales of goods’ COM (2015) 635 final.

75 See eg Reiner Schulze, Dirk Staudenmayer and Sebastian Lohsse (eds), *Contracts for the Supply of Digital Content: Regulatory Challenges and Gaps*: *Münster Colloquia on EU Law and the Digital Economy II* (Nomos 2017); European Law Institute (ELI), *Statement on the European Commission’s Proposed Directive on the Supply of Digital Content to Consumers COM (2015) 634 final* (ELI 2016). Many commentators, especially in Germany, have participated in the debate. A partial list includes Philipp Hacker, ‘Daten als Gegenleistung: Daten als Gegenleistung. Rechtsgeschäfte im Spannungsfeld von DS-GVO und allgemeinem Vertragsrecht’ [2019] Zeitschrift für die gesamte Privatrechtswissenschaft 14; Niko Härting, ‘Digital Goods und Datenschutz – Daten sparen oder monetarisieren? Die Reichweite des vom DinhRL-E erfassten Geschäftsmodells’ (2016) 11 Computer und Recht 735; Axel Metzger, ‘Data as Counter-Performance – What Rights and Duties do Parties Have?’ (2017) 8 Journal of Intellectual Property, Information Technology and E-Commerce Law 2; Andreas Sattler, ‘Personenbezogene Daten als Leistungsgegenstand – Die Einwilligung als Wegbereiter des Datenschuldrechts’ (2017) 72 Juristenzeitung 1036; Martin Schmidt-Kessel and others, ‘Die Richtlinienvorschläge der Kommission zu Digitalen Inhalten und Online-Handel: Teil 1’ [2016] Zeitschrift für das Privatrecht der Europäischen Union 2; Martin Schmidt-Kessel and others, ‘Die Richtlinienvorschläge der Kommission zu Digitalen Inhalten und Online-Handel: Teil 2’ [2016] Zeitschrift für das Privatrecht der Europäischen Union 54; Louisa Specht, ‘Daten als Gegenleistung – Verlangt die Digitalisierung nach einem neuen Vertragstypus?’ (2017) 72 Juristenzeitung 763; Gerald Spindler, ‘Verträge über digitale Inhalte – Anwendungsbereich und Ansätze – Vorschlag der EU-Kommission zu einer Richtlinie über Verträge zur Bereitstellung digitaler Inhalte’ (2016) 19 Multimedia und Recht 147; Gerald Spindler, ‘Verträge über digitale Inhalte – Haftung, Gewährleistung und Portabilität – Vorschlag der EU-Kommission zu einer Richtlinie über Verträge zur Bereitstellung digitaler Inhalte’ (2016) 19 Multimedia und Recht 219; Friedrich Graf von Westphalen, ‘Richtlinienentwurf der Kommission betreffend die Bereitstellung digitaler Inhalte und das Recht des Verbrauchers auf Schadensersatz’ [2016] Betriebsberater 1411; Friedrich Graf von Westphalen and Christiane Wendehorst, ‘Hergabe personenbezogener Daten für digitale Inhalte – Gegenleistung, bereitzustellendes Material oder Zwangsbeitrag zum Datenbinnenmarkt?’ [2016] Betriebsberater 2179.

76 For the draft of the Digital Content Directive, see Council, ‘Proposal for a Directive of the European Parliament and of the Council on certain aspects concerning contracts for the supply of digital content (First reading) – General approach’ 9901/17 ADD 1, 2015/0287 (COD), 01.06.2017 (hereinafter referred to as ‘Council-DCD’); European Parliament, ‘Report on the proposal for a directive of the European Parliament and of the Council on certain aspects concerning contracts for the supply of digital content (COM(2015)0634 – C8-0394/2015 – 2015/0287(COD))’ A8-0375/2017, 27.11.2017 (hereinafter referred to as ‘EP-DCD’).

77 See Nikolina Sajn, ‘Contracts for the Supply of Digital Content and Digital Services’ (PE 635.601, March 2019) <www.europarl.europa.eu/RegData/etudes/BRIE/2019/635601/EPRS_BRI(2019)635601_EN.pdf> accessed 21 February 2020.

78 Parliament and Council Directive (EU) 2019/770 of 20 May 2019 on certain aspects concerning contracts for the supply of digital content and digital services, OJ L136/1 (hereinafter referred to as ‘DCSD’); Parliament and Council Directive (EU) 2019/771 of 20 May 2019 on certain aspects concerning contracts for the sale of goods, amending Regulation (EU) 2017/2394 and Directive 2009/22/EC, and repealing Directive 1999/44/EC, OJ L136/28 (hereinafter referred to as ‘SGD’).

79 These issues received an earlier treatment *inter alia* in Axel Metzger and others, ‘Data-Related Aspects of the Digital Content Directive’ (2018) 9 Journal of Intellectual Property, Information Technology and E-Commerce Law 90.

80 DCSD, recital 11.

81 COM-DCD, art 3(1) and recitals 13, 14.

82 EDPS, ‘Opinion 4/2017 on the Proposal for a Directive on certain aspects concerning contracts for the supply of digital content’ (EDPS 14 March 2017) <https://edps.europa.eu/sites/edp/files/publication/17-03-14_opinion_digital_content_en.pdf> accessed 21 February 2020.

83 Note that the DCSD (as opposed to the COM-DCD) no longer includes the phrase ‘counter-performance’ in connection with personal data provided by the consumer. It further omitted the phrase ‘in exchange’, which referred in the COM-DCD directly to the phrase ‘a consumer actively provid[ing] counter-performance other than money in the form of personal data or any other data’.

84 DCSD, art 3(1).

85 DCSD, art 3(8).

86 ibid.

87 cf Carmen Langhanke and Martin Schmidt-Kessel, ‘Consumer Data as Consideration’ (2015) 6 EuCML 218, 219ff. Here it is suggested that two ‘layers’ of consumer protection apply in such cases.

88 COM-DCD, recital 14: ‘As regards digital content supplied not in exchange for a price but against counter-performance other than money, this Directive *should apply only* to contracts where the supplier requests and the consumer *actively* provides data’ (emphasis added).

89 Council-DCD, art 3(1) at n 15.

90 ibid at n 15.

91 EP-DCD, recital 14. This Recital also mentioned as covered by the directive ‘the name and e-mail address or photos, provided directly or indirectly to the trader, for example through individual registration or on the basis of a contract which allows access to consumers’ photos’. ibid.

92 See eg Metzger and others (n 79) 96, paras 26-28; Spindler, ‘Verträge über digitale Inhalte – Anwendungsbereich und Ansätze’ (n 75) 149-159; Hacker (n 75) 166.

93 cf Graf von Westphalen and Wendehorst (n 75) 2180-2181.

94 DCSD, recital 25: ‘This Directive should also not apply to situations where the trader only collects metadata, such as information concerning the consumer’s device or browsing history, except where this situation is considered to be a contract under national law […]. However, Member States should remain free to extend the application of this Directive to such situations, or to otherwise regulate such situations, which are excluded from the scope of this Directive.’

95 cf Axel Metzger, ‘A Market Model for Personal Data: State of the Play under the New Directive on Digital Content and Digital Services’ in Sebastian Lohsse, Reiner Schulze and Dirk Staudenmayer (eds), *Data as Counter Performance – Contract Law 2.0? Münster Colloquia on EU Law and the Digital Economy V* (Nomos, 2020) (forthcoming) 3-4 <www.rewi.hu-berlin.de/de/lf/ls/mzg/pub/metzger-markets-for-data-oct-2019-preprint.pdf> accessed 21 February 2020.

96 DCSD, recital 24: ‘The personal data could be provided to the trader either at the time when the contract is concluded or at a later time, such as when the consumer gives consent for the trader to use any personal data that the consumer might upload or create with the use of the digital content or digital service.’

97 For more discussion, see Zohar Efroni, ‘Gaps and Opportunities: The Rudimentary Protection to “Data-Paying Consumers” under New EU Consumer Protection Law’ 57(3) Common Market Law Review 799-830 (2020).

98 For a comprehensive treatment and argument that metadata is more than merely ‘data about data’, see Jeffrey Pomerantz, *Metadata* (MIT Press 2015).

99 cf Gerald Spindler and Karin Sein, ‘Die endgültige Richtlinie über digitale Inhalte und Dienstleistungen’ [2019] Multimedia und Recht 415, 418. Here DCSD, recital 25 is read as excluding cookies information generally, and cookies information that qualifies as personal data specifically.

100 This outcome was criticised, eg, by Lena Mischau, ‘Daten als “Gegenleistung” im neuen Verbrauchervertragsrecht’ [2020] Zeitschrift für die gesamte Privatrechtswissenschaft 5 (forthcoming).

101 For simplicity, we set aside the second exception in DCSD, art 3(1) regarding data processed in order to comply with a legal obligation.

102 GDPR, art 4(1): ‘“Personal data” means any information relating to an identified or identifiable natural person (“data subject”); an identifiable natural person is one who can be identified, directly or indirectly, in particular by reference to an identifier such as a name, an identification number, location data, an online identifier or to one or more factors specific to the physical, physiological, genetic, mental, economic, cultural or social identity of that natural person.’

103 Case C-582/14 *Breyer* [2016] EU:C:2016:779. This judgment refers to the definition of ‘personal data’ under Parliament and Council Directive (EC) 95/46 of 24 October 1995 on the protection of individuals with regard to the processing of personal data and on the free movement of such data [1995] OJ L281/31, art 2(a), which is considered equivalent to the definition of the same concept under the GDPR.

104 For a recent account, see Michèle Finck and Frank Pallas, ‘They Who Must Not Be Identified – Distinguishing Personal from Non-Personal Data under the GDPR’ (2019) Max Planck Institute for Innovation and Competition Research Paper No. 19-14 <https://papers.ssrn.com/sol3/papers.cfm?abstract_id=3462948> accessed 21 February 2020.

105 COM (2017) 10 final: ePrivacy Regulation Proposal, recital 17.

106 cf Finck and Pallas (104) 30-31. See also, Keßler and McKenzie (n 14).

107 DCSD, arts 5-8.

108 European Data Protection Board, ‘Guidelines 2/2019 on the processing of personal data under art 6(1)(b) GDPR in the context of the provision of online services to data subjects’ (v. 2.0, 8 October 2019) para 22.

109 GDPR, art 6(1)(a). Legitimate interests under GDPR, art 6(1)(f) can provide an alternative ground.

110 Case C-673/17 *Planet49 GmbH v. Bundesverband der Verbraucherzentralen und Verbraucherverbände – Verbraucherzentrale Bundesverband e.V.* [2019] EU:C:2019:246, paras 49-65.

111 ibid paras 70-71.

112 ibid para 56.

113 Amended Proposal of the ePrivacy Regulation, art 6bff. Shortly before this chapter went to print, the Presidency of the Council of the European Union published a progress report regarding the ePrivacy Regulation Proposal. The Presidency proposed a modification, according to which the processing of electronic communication metadata would be allowed also when it is necessary for the purpose of legitimate interests (i.e., without specific user consent), subject to a number of safeguards. See Council of the European Union, Proposal for a Regulation of the European Parliament and of the Council concerning the respect for private life and the protection of personal data in electronic communications and repealing Directive 2002/58/EC (Regulation on Privacy and Electronic Communications) - Progress Report, 29 May 2020 <https://data.consilium.europa.eu/doc/document/ST-8204-2020-INIT/en/pdf> accessed 26 August 2020.

114 One way to combat lack of awareness, risk illiteracy and information asymmetry for achieving a better working consent procedure is through visualisation techniques such as privacy icons. See eg Zohar Efroni and others, ‘Privacy Icons: A Risk-Based Approach to Visualisation of Data Processing’ (2019) 3 European Data Protection Law Review 352. Another way is by applying personal information management systems (PIMS). See EDPS, ‘Opinion on Personal Information Management Systems (Opinion 9/2016): Towards more user empowerment in managing and processing personal data’ (EDPS 2016) 5 <https://edps.europa.eu/sites/edp/files/publication/16-10-20_pims_opinion_en.pdf> accessed 21 February 2020; Stiftung Datenschutz, ‘Policy Paper: New Ways of Providing Consent in Data Protection – Technical, Legal and Economic Challenges’ (Stiftung Datenschutz 2017) <https://stiftungdatenschutz.org/fileadmin/Redaktion/Bilder/Abschluss_Studie_30032017/stiftungdatenschutz_PolicyPaper_New_ways_of_providing_consent_in_data_protection_EN_final.pdf> accessed 21 February 2020.

115 DCSD, art 2(3).

116 For an extensive discussion on the concept and meanings of IoT, see Ovidiu Vermesan and others, ‘Internet of Things – Strategic Research and Innovation Agenda’ in Ovidiu Vermesan and Peter Friess (eds), *Internet of Things* (River Publishing 2014) 11-16.

117 ibid 12: ‘In [the IoT] context the notion of network convergence using IP is fundamental and relies on the use of a common multi-service IP network supporting a wide range of applications and services. The use of IP to communicate with and control small devices and sensors opens the way for the convergence of large, IT-oriented networks with real time and specialised networked applications.’

118 DCSD, recital 21: ‘Directive (EU) 2019/771 [SGD] should apply to contracts for the sale of goods, including goods with digital elements. The notion of goods with digital elements should refer to goods that incorporate or are inter-connected with digital content or a digital service in such a way that the absence of that digital content or digital service would prevent the goods from performing their functions. Digital content or a digital service that is incorporated in or inter-connected with goods in that manner should fall within the scope of Directive (EU) 2019/771 if it is provided with the goods under a sales contract concerning those goods. Whether the supply of the incorporated or inter-connected digital content or digital service forms part of the sales contract with the seller should depend on the content of this contract.’

119 SGD, art 3.1.

120 SGD, art 2(1): ‘“Sales contract” means any contract under which the seller transfers or undertakes to transfer ownership of goods to a consumer, and the consumer pays or undertakes to pay the price thereof.’

121 DCSD, recital 21.

122 SGD, art 7(3).

123 For more discussion, see Axel Metzger, ‘Verträge über digitale Inhalte und digitale Dienstleistungen: Neuer BGB-Vertragstypus oder punktuelle Reform?’ (2019) 12 Juristenzeitung 577, 578.

124 A random list of the ‘most popular’ IoT devices shows that all the products on the list are currently offered for money—some for a considerable price. See ‘18 Most Popular IoT Devices in 2020 (Only Noteworthy IoT Products)’ (*Software Testing Helps*, 25 December 2019) <www.softwaretestinghelp.com/iot-devices> accessed 21 February 2020.

125 Parliament and Council Directive (EU) 2019/2161 of 27 November 2019 amending Council Directive 93/13/EEC and Directives 98/6/EC, 2005/29/EC and 2011/83/EU of the European Parliament and of the Council as regards the better enforcement and modernisation of Union consumer protection rules [2019] OJ L328/7.

126 CRMD, art 4(1). See referenced and new definitions in the revised CRD to ‘goods’, ‘personal data’, ‘sales contract’, ‘service contract’, ‘digital content’ and ‘digital service’ as well as to ‘computability’, ‘functionality’ and ‘interoperability’.

127 CRMD, art 4(2)(b).

128 CRD, art 5(1)(g)-(h).

129 CRD, art 9(1), 9(2)(c).

